# Physiological platelet aggregation assay to mitigate drug-induced thrombocytopenia using a microphysiological system

**DOI:** 10.1038/s41598-024-64063-y

**Published:** 2024-06-19

**Authors:** Kosuke Harada, Wang Wenlong, Tadahiro Shinozawa

**Affiliations:** Drug Safety Research and Evaluation, Research, Takeda Pharmaceutical Company Limited, 26-1, Muraoka-Higashi 2-Chome, Fujisawa, Kanagawa 251-8555 Japan

**Keywords:** Drug discovery, Physiology

## Abstract

Developing a reliable method to predict thrombocytopenia is imperative in drug discovery. Here, we establish an assay using a microphysiological system (MPS) to recapitulate the in-vivo mechanisms of platelet aggregation and adhesion. This assay highlights the role of shear stress on platelet aggregation and their interactions with vascular endothelial cells. Platelet aggregation induced by soluble collagen was detected under agitated, but not static, conditions using a plate shaker and gravity-driven flow using MPS. Notably, aggregates adhered on vascular endothelial cells under gravity-driven flow in the MPS, and this incident increased in a concentration-dependent manner. Upon comparing the soluble collagen-induced aggregation activity in platelet-rich plasma (PRP) and whole blood, remarkable platelet aggregate formation was observed at concentrations of 30 µg/mL and 3 µg/mL in PRP and whole blood, respectively. Moreover, ODN2395, an oligonucleotide, induced platelet aggregation and adhesion to vascular endothelial cells. SYK inhibition, which mediated thrombogenic activity via glycoprotein VI on platelets, ameliorated platelet aggregation in the system, demonstrating that the mechanism of platelet aggregation was induced by soluble collagen and oligonucleotide. Our evaluation system partially recapitulated the aggregation mechanisms in blood vessels and can contribute to the discovery of safe drugs to mitigate the risk of thrombocytopenia.

## Introduction

Platelets prevent bleeding in the body. When a blood vessel is injured, platelets attach to the wound and aggregate to form a primary thrombus, playing a major role in hemostasis^1^. Platelets are the second most common blood cell component after red blood cells; in humans, 150,000–450,000 platelets are present in 1 µL of blood^2^. However, hemostasis does not function normally when the platelet count decreases, resulting in death in severe cases^3^. A decrease in platelet counts is an adverse effect in drug development. Various drugs cause thrombocytopenia, and a decrease in platelets has been observed in recently marketed oligonucleotide drugs^4–6^. Therefore, evaluating the risk of thrombocytopenia is crucial in early drug discovery stages to avoid severe adverse reactions in clinical settings. Several mechanisms reduce the platelet count, and aggregation via drug-induced platelet activation is a cause of decreased platelet counts^7,8^.

Light transmission aggregometry is generally used as a gold standard for assessing platelet function^9,10^, in which platelet-rich plasma is stirred in a test tube and the test article reacts^9^. However, in vivo, blood flows through vessels lined with vascular endothelial cells. The mode of shear stress on platelets and the presence of vascular endothelial cells affect platelet aggregation activity^11–13^. Platelet aggregometry assays are performed without considering factors that play crucial roles in thrombosis, such as fluid hemodynamics and vascular endothelial cells. Therefore, to accurately evaluate the platelet aggregation potential, it is necessary to develop a method recapitulating the vascular environment, in which fluid hemodynamics and vascular endothelial cells are present. Jain et al.^11^ successfully perfused whole blood into a tube of vascular endothelial cells by using an organ-on-chip system to observe platelet aggregates against vascular endothelial cells^11^. This sophisticated model is informative because it can recapitulate hemodynamic function in vitro and evaluate its effect on platelets in an environment that includes vascular endothelial cells, blood flow, and whole blood. However, this microphysiological system (MPS), which adopts an active pumping mechanism using peristaltic and syringe pumps, has a low throughput, complicating the evaluation of multiple samples. Handling this test system is also difficult for researchers unfamiliar with MPS testing. Recently, an MPS that uses gravity-driven flow has attracted considerable attention^14,15^. Although adjusting the flow rate of a gravity-driven flow system is difficult, a fixed flow rate can easily be achieved using a plate rocker, and multiple samples can be evaluated by placing the MPS on such a device.

In this study, we developed a novel platelet aggregation assay system using MPS to evaluate the platelet aggregation potential in the presence of vascular endothelial cells and shear stress, essential factors in thrombosis. In addition, using this model, we examined whether the MPS platelet aggregation assay could evaluate oligonucleotide-induced platelet aggregation.

## Results

### Effect of perfusion method on platelet aggregation and adhesion on vascular endothelial cells

**Figure 1:**
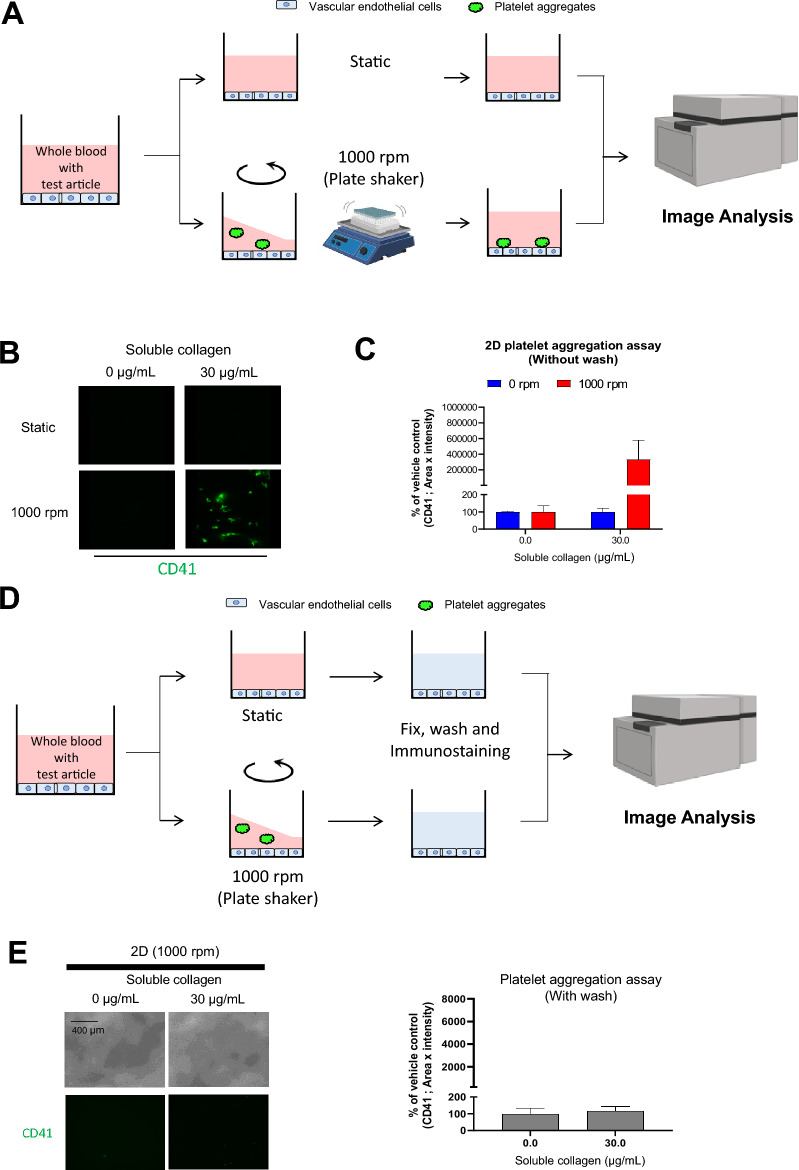
Platelet aggregation assay using whole blood from mice in the 2D culture model. (**A**) Schematic representation of the platelet aggregation assay, without washing, in the 2D culture model. (**B**) Fluorescent images of CD41 (green) and (**C**) fluorescent intensity analysis in the platelet aggregation assay, without washing, in the 2D culture model. Data are presented as means ± SD (n = 4). (**D**) Schematic representation of the platelet aggregation assay, with washing, in the 2D culture model. (**E**) Phase contrast and fluorescent images of CD41 (green) in the platelet aggregates adhering to vascular endothelial cells. The fluorescent intensity in the platelet aggregation assay, with washing in the 2D culture model, is also shown. Data are presented as means ± SD (n = 3).

**Figure 2:**
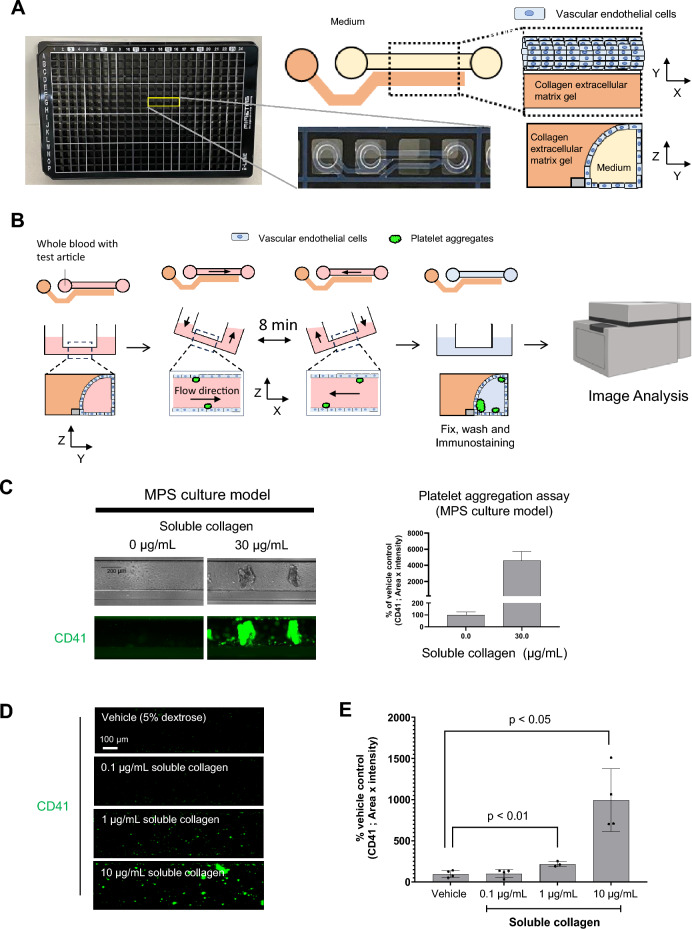
Platelet aggregation assay using murine whole blood in the MPS culture model. (**A**) Schematic representation of the microphysiological system (MPS) culture model in the two-lane OrganoPlate platform, and (**B**) the platelet aggregation assay, with washing, in the MPS culture models. (C) Phase contrast and fluorescent images of CD41 (green) in the platelet aggregates adhering to vascular endothelial cells. Fluorescent intensities in the platelet aggregation assay, with washing in the MPS culture models, is also shown. Data are presented as means ± SD (n = 3). (**D**) Representative fluorescent images of CD41 (green) and (**E**) fluorescent intensity analysis in concentration–response experiments using collagen in the platelet aggregation assay in the MPS culture model. Data are presented as means ± SD (n = 3). Student’s t-test was used to compare treated to vehicle-treated conditions.

We first examined the effect of perfusion on platelet aggregate formation and the interaction with vascular endothelial cells in whole blood using soluble collagen, which is commonly used as a positive control in platelet aggregometry assays. Soluble collagen was added to whole blood and stirred to compare the conditions without stirring. The agitation speed was set based on the assay condition of 96 well plate platelet aggregation test (LTA assay) as previously reported^16^. Platelet aggregates was just observed with agitation at 1000 rpm (Fig. 1A–C). However, no aggregates were observed on the vascular endothelial surface when the whole blood was washed with phosphate-buffered saline (PBS) after incubation (Fig. 1D,E). In contrast, when a vascular endothelial tube was formed in the organoplate, and soluble collagen and whole blood were incubated with flow, platelet aggregate adhesion on the vascular endothelial cells was observed even after washing the channel with PBS (Fig. 2A–C). In the test using soluble collagen, platelet marker CD41-positive aggregates were formed more prominently in the soluble collagen ≥ 1 µg/mL groups than in the vehicle group (Fig. 2D,E).

### Comparison between whole blood and platelet-rich plasma for platelet aggregate formation in MPS platelet aggregation assay

**Figure 3:**
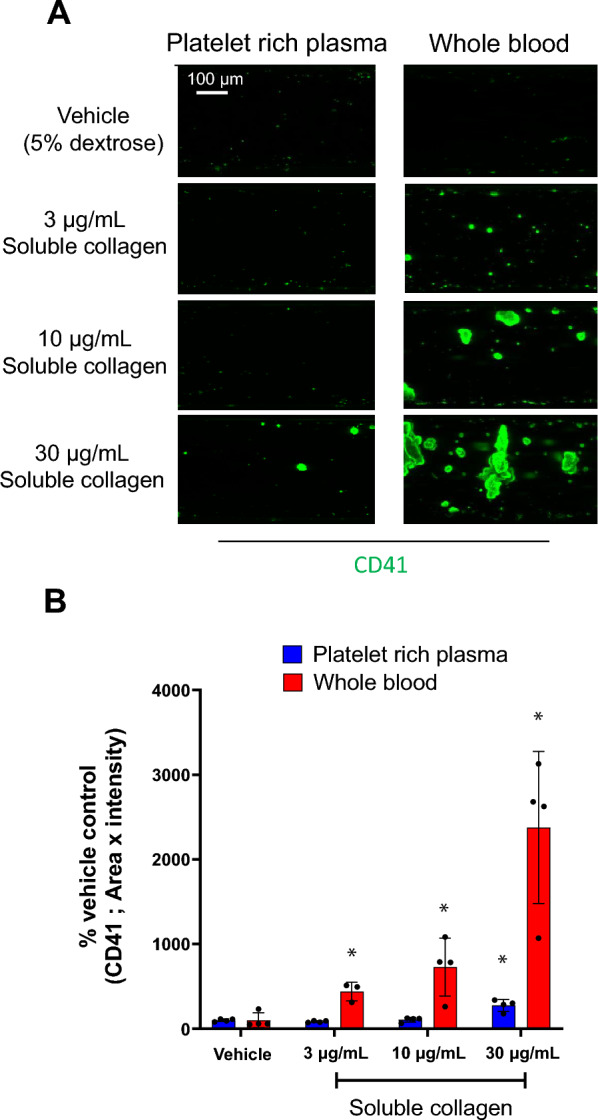
Comparison between platelet-rich plasma and whole blood using the MPS culture model in the platelet aggregation assay. (**A**) Representative fluorescent images of CD41 (green) in the channels were exposed to murine platelet-rich plasma and whole blood. (**B**) Fluorescent intensity analysis of the platelet aggregation assay using murine platelet-rich plasma (blue) and whole blood (red). Data are presented as the mean ± SD (n = 3). Student’s t-test was used to compare vehicle treatment conditions. Statistical significance is presented relative to the vehicle-treated conditions as *P < 0.05.

Blood cell components other than platelets are also involved in aggregate formation^17,18^. However, conventional platelet aggregation assays primarily use platelet-rich plasma (PRP)^9^ and do not reflect the effects of blood cells other than platelets and other blood components included in whole blood. Therefore, to investigate the sensitivity of aggregate formation in whole blood, we compared the reactivities of PRP and whole blood during soluble collagen treatment on the MPS platform. Soluble collagen induced significant platelet aggregation at 30 µg/mL and 3 µg/mL in PRP and whole blood, respectively (Fig. 3A,B).

### Inhibition test of platelet aggregates formation using a SYK pathway inhibitor in MPS platelet aggregation assay

**Figure 4:**
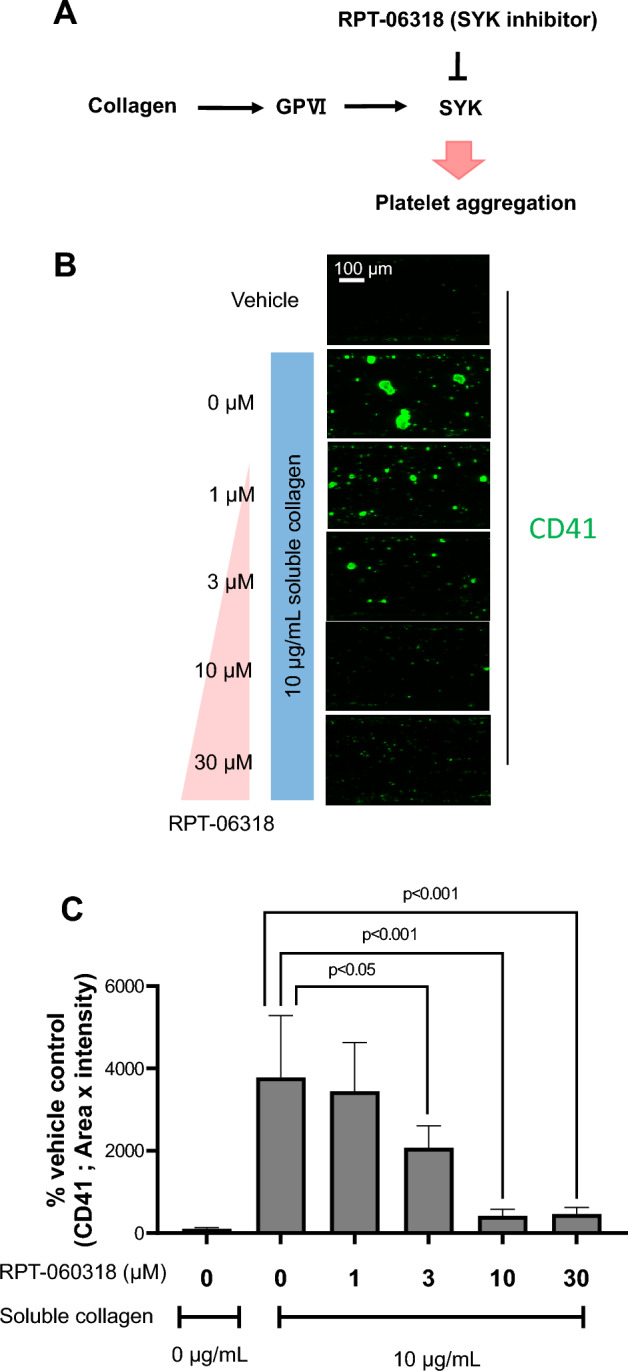
Effect of SYK inhibition on platelet aggregate formation in soluble collagen treated with whole blood in the MPS culture model. (**A**) Schematic representation of suppression of platelet aggregation by the SYK inhibitor. (**B**) Representative fluorescent images of CD41 (green) in the channels exposed to mice whole blood. (**C**) Fluorescent intensity analysis of SYK inhibition on platelet aggregate formation in mice whole blood. Data are presented as the mean ± SD (n = 8). Student’s t-test was used to compare 10 µg/mL of soluble collagen treatment conditions.

Collagen binds to GPVI expressed on the platelet surface and activates downstream pathways, which activates platelets and causes platelet aggregation^19–21^. SYK is downstream of GPVI signaling, and its inhibition can inhibit platelet aggregation mediated by the GPVI pathway (Fig. 4A). Soluble collagen and the SYK inhibitor RPT-060318 were co-treated to verify whether the formation of soluble collagen-induced platelet aggregates detected by the MPS platelet aggregation assay was caused by a mechanism similar to that of another report^21^. SYK inhibitor significantly inhibited collagen-induced platelet aggregation at ≥ 3 µM (Fig. 4B,C).

### Evaluation of oligonucleotide induced platelet aggregation using the MPS platelet aggregation assay

**Figure 5:**
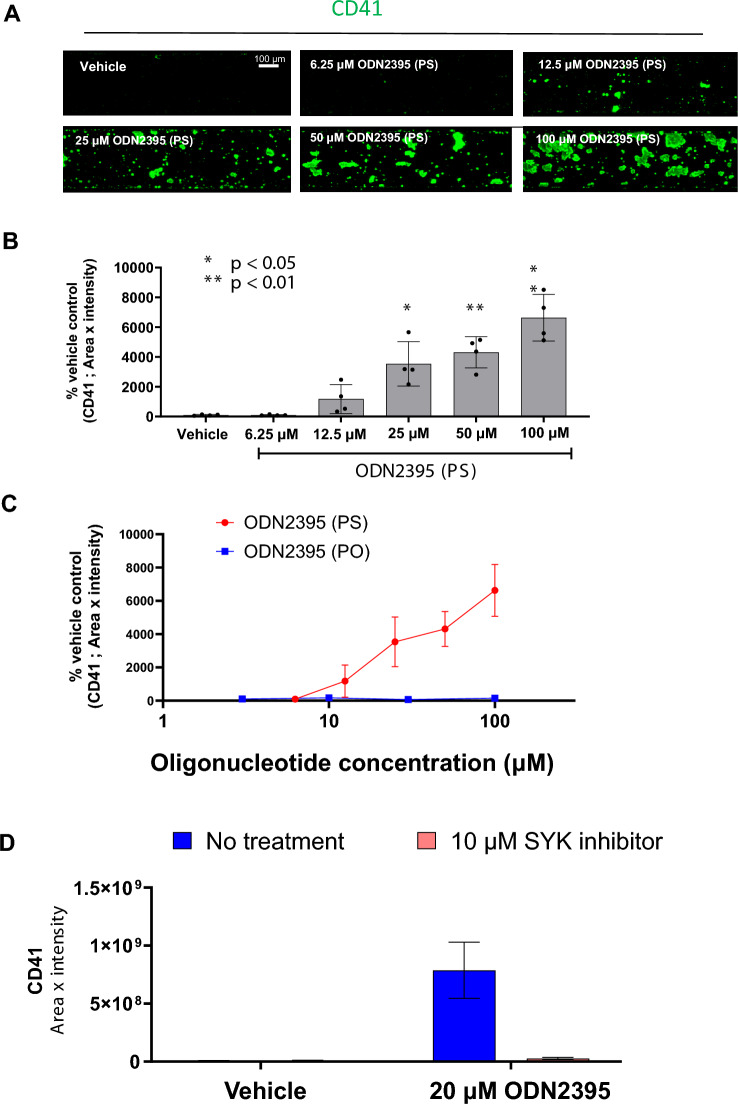
Effect of oligonucleotides on platelet aggregate formation in the MPS culture model. (**A**) Representative fluorescent images of CD41 (green) (**B**) and fluorescent intensity analysis in dose–response experiments of ODN2395 phosphorothioate backbone (PS)-induced platelet aggregation. Data are presented as the mean ± SD (n = 4). Student’s t-test was used to compare vehicle treatment conditions. Statistical significance is presented relative to the vehicle-treated conditions as *P < 0.05, **P < 0.01. (**C**) Effect of modifying the oligonucleotide backbone on platelet aggregate formation. Data are presented as the mean ± SD (n = 4). (**D**) Fluorescent intensity analysis of the effect of SYK inhibition on platelet aggregate formation in ODN2395 PS-treated whole blood. Data are presented as the mean ± SD (n = 8).

GPVI protein-mediated platelet activation occurs in oligonucleotide therapeutics because oligonucleotides have a charge and structure similar to those of collagen^19^. Therefore, ODN2395, with a phosphorothioate (PS) backbone, which activates and aggregates platelets, was evaluated using the MPS platelet aggregation assay. We detected platelet aggregate formation in ODN2395 with a PS backbone similar to collagen (Fig. 5A,B). However, no platelet aggregation activity was observed under conditions in which the ODN2395 backbone was replaced with a phosphodiester (PO) backbone to reduce protein-binding activity (Fig. 5C). Furthermore, after treatment with an SYK inhibitor, no aggregate formation by ODN2395 with the PS backbone was observed (Fig. 5D).

## Discussion

Conventional platelet functional assays, such as light transmission aggregometry, the gold standard for assessing platelet function, have low throughput and require specialized equipment. In contrast, our evaluation system improved the throughput by combining gravity-driven flow with a high-content imaging system, enabling the evaluation of platelet aggregation activity in multiple samples. Further studies verifying high-throughput implementation could contribute to confirmation of the feasibility. Conventional platelet aggregation assays assess platelet aggregation directly using PRP via light transmission aggregometry or indirectly using whole blood via impedance aggregometry^22^. However, interactions with blood flow and vascular endothelial cells are also significantly involved in platelet aggregation in vivo, in addition to blood cells and plasma components^11–13^. In this study, we used an MPS to develop a novel platelet aggregation assay that recapitulates the in-vivo environment in the presence of blood, vascular endothelial cells, and blood flow.

Soluble collagen is widely used as a physiological agonist to measure platelet aggregation^23^. The aggregation activity of soluble collagen was evaluated using a 2D culture model of vascular endothelial cells. When soluble collagen and whole blood were added to this model and statically incubated, platelet aggregation was not observed in whole blood. However, when whole blood with soluble collagen was stirred, platelet aggregates formed. The reason for the absence of aggregate formation under static conditions was that mechanical shear stimulation played a dominant role in platelet aggregation^24^. The agglutination test was performed by stirring in the conventional platelet aggregation assay^25^, suggesting that creating an environment in which platelets are stimulated by mechanical shear stress to evaluate platelet aggregate formation in the in-vitro evaluation system is essential. No platelet aggregates were detected when whole blood treated with soluble collagen was stirred and washed in the 2D culture model. Presumably, platelet aggregates were present as suspensions in the whole blood and were removed by washing with PBS after incubation. When the same test was performed in the MPS model, platelet aggregates adhered to the surface of vascular endothelial cells after washing with PBS. In addition, the number of platelet aggregates adhering to vascular endothelial cells increased in a concentration-dependent manner after soluble collagen treatment.

The difference in the adhesion of platelet aggregates to vascular endothelial cells observed between the 2D and MPS models may have been affected by the perfusion method of whole blood. The flow in the orbital shaker was directed toward the sidewalls of the well and slightly toward the liquid surface^26^. Therefore, interactions between platelets and vascular endothelial cells in an orbital shaker may hardly occur. In contrast, in the MPS, the environment is likely to allow platelet adhesion to the vascular endothelial cell surface, similar to blood flow in the body^26–28^.

Under laminar flow in blood vessels, the red blood cells flow into the center of the blood vessel and push platelets toward the blood vessel wall (Supplementary Fig. 1A), which is termed platelet margination^29^. By providing laminar flow within the endothelial tube in the MPS chamber, we hypothesized that platelet margination could lead to platelet adhesion onto the surface of vascular endothelial cells (Supplementary Fig. 1B). Another possibility is that the function of vascular endothelial cells differs between 2D and shear-stressed MPS cultures. When cells isolated from living organisms are cultured in a static, 2D manner, their inherent functions are lost^30^. However, the MPS can maintain the function of cells by imitating the environment in vivo compared to 2D culture^31^. Mechanical stimulation by shear stress is converted to intracellular signals in vascular endothelial cells, affecting cellular functions involved in homeostatic functions, including hemostasis and thrombosis^29,32,33^. Therefore, when vascular endothelial cells are cultured under flow conditions, mechanical stimulation may improve their homeostatic function, and the adhesion of platelet aggregates can be detected only in the MPS model.

The adhesion of platelet aggregates on vascular endothelial cells observed in this study is challenging to evaluate via the platelet aggregation potential using conventional assay systems. In a conventional evaluation system, PRP reacts in a plastic tube, and the ability to form aggregates is indirectly evaluated as a decrease in turbidity when platelet aggregates are formed^9,25^. However, in our evaluation system, the aggregation activity of platelets was evaluated under conditions mimicking blood components and the vascular endothelium and blood flow. Therefore, the aggregation activity of platelets and the adhesion of aggregates to vascular endothelial cells could be evaluated.

PRP is used to measure platelet aggregation activity^9^. Although the PRP method can evaluate the direct effect on platelets, blood cell components other than platelets are involved in platelet activation and aggregate formation as well^17,18^. Therefore, we compared PRP and whole blood as MPS platelet aggregation assay materials. The platelet aggregation activity was significantly higher than that of PRP using whole blood, and platelet aggregates were formed in the soluble collagen-treated group at a lower concentration than those in the PRP group. Various cytokines and platelet activators are secreted when platelets are activated, thereby activating immune cells^18,34^. Activated immune cells activate platelets by binding to them, resulting in accelerated platelet activation and aggregate formation^35^.

GPVI, a collagen receptor expressed on the surface of platelets, binds collagen to GPVI and activates platelets via the GPVI-induced signaling pathway^36^. Many molecules are involved in the GPVI induced signaling pathway; however, SYK, a protein tyrosine kinase, specifically plays a central role in the GPVI-induced signaling pathway and activates platelets by increasing the calcium ion concentration in platelets^36^. SYK inhibitors inhibit platelet activation and aggregation by inhibiting SYK-mediated phosphorylation^37^.

Platelet aggregate formation in our MPS platelet aggregation assay was inhibited by the SYK inhibitor, indicating that the GPVI pathway could mediate soluble collagen-induced platelet aggregation. These results indicate that the aggregation activity of a compound can be evaluated by quantifying the amount of platelet aggregation using the MPS platelet aggregation assay. This evaluation system can also evaluate platelet aggregation inhibition by a compound.

In oligonucleotide therapeutics, which have recently attracted attention, the PS backbone has often been used to prevent nuclease activity, which is involved in oligonucleotide degradation^38^. However, oligonucleotides with a PS backbone have higher protein-binding activity than those with a PO backbone, such as collagen; they bind to GPVI receptors to activate them, thus activating platelets^19,21,38,39^. In our evaluation system, when ODN2395 with a PS backbone was used, we observed platelet aggregate formation and adhesion to vascular endothelial cells. However, when the oligonucleotide backbone was exchanged with a PO backbone, no platelet aggregate formation was observed, which is consistent with previous studies^19^. The formation of platelet aggregates induced by ODN2395 with a PS backbone was inhibited by SYK inhibitors, indicating that the our MPS platelet aggregation assay could detect GPVI receptor-mediated platelet aggregation induced by ODN2395, as previously reported^21^.

Severe drug-induced thrombocytopenia is a serious adverse event that can lead to life-threatening conditions. Several mechanisms reduce the platelet count, and platelet aggregation by drugs is one cause of a decreased platelet count^7,8^. We established a novel system for evaluating platelet aggregation using the MPS. The assay evaluates the ability of platelets to form aggregates, including interactions between platelets and vascular endothelial cells, which are challenging to detect using conventional assay systems. This evaluation system can contribute to developing safer drugs by evaluating platelet aggregation potentials, including interactions between vascular endothelial cells and platelets. Future studies should investigate the relationship between aggregate formation of immune cells.

## Materials and methods

### Chemicals and antibodies

Reconstitution buffer (635-00791) was purchased from Nitta Gelatin (Osaka, Japan). Albumin solution (30w/v%) and bovine serum albumin (BSA; 017-22231) were purchased from FUJIFILM Wako Pure Chemical Corporation, Osaka, Japan. ODN2395 was chemically synthesized and purified by GeneDesign, Inc. (Ibaraki, Japan). RPT-060318 (S7738) was purchased from Selleck Chemicals LLC (Houston, TX, USA). Soluble collagen (AW993826) was purchased from Sysmex Corporation (Hyogo, Japan).

### Cell culture in 96-well culture plates

Cell culture plates (96-well, Falcon, New York, NY, USA) were treated with GLS250 gelatin solution (100 µL/well; 630-36795; Nitta Gelatin, Osaka, Japan) at 37 °C for 1 h. The plates were washed with 10× PBS (163-25265; FUJIFILM Wako Pure Chemical Corporation, Osaka, Japan). Murine brain microvascular endothelial cells were seeded (4000 cells/well) in gelatin-coated 96-well plates in basal medium supplemented with VEGF (M1168; Cell Biologics). The medium was changed every 2–3 days. On day 7, the cells were used for the platelet aggregation assay.

### Cell culture in OrganoPlate® 2-lane

Collagen extracellular matrix (ECM) gel was prepared by mixing Cellmatrix Type 1-A (631-00651; Nitta Gelatin, Osaka, Japan), preparation buffer, and 10× PBS at a ratio of 8:1:1. Collagen ECM gel (3 µL) was dispensed into the ECM gel inlet. The OrganoPlate® 2-lane (9605-400-B; Mimetas, Leiden, the Netherlands) was placed in a humidified incubator for 30 min. PBS (50 µL) was added to the ECM gel inlet. Then, 50,000–100,000 cells/mL murine brain microvascular endothelial cell suspensions (C57-6023; Cell Biologics, Inc, Chicago, IL, USA) were prepared. Cell suspension (2 µL) was seeded in the medium inlet. The OrganoPlate® 2-lane was placed in a humidified incubator for 4 h. After incubation, the medium (50 µL) was added to the medium inlet. The OrganoPlate® 2-lane was placed on an interval rocker, switching between a + 7° and − 7°inclinations every 8 min in a humidified incubator. The medium was changed every 2–3 days. On day 7, the cells were used for the platelet aggregation assay.

### Preparation of mice whole blood

Murine whole blood collection was performed at Jackson Laboratory following the guidelines of the Animal Ethics Committee at that laboratory, which were established independently of the research members according to the guidance of the Japanese Society for laboratory animal resources or AAALAC international.

### Platelet aggregation assay without washing in the 2D culture model

Murine whole blood (Jackson Laboratory, Tokyo, Japan) was treated with APC-anti-CD41 antibodies (1/100 volume; 133914; BioLegend, San Diego, CA, USA) and incubated at 25 °C for 20 min. The medium was removed from the 96-well cell culture plates, and the cells were washed with PBS. Then, whole blood (80 µL/well) pre-stained with APC-anti-CD41 antibody was dispensed into 96-well cell culture plates, and an evaluation compound (20 µL) was added. After incubation at 37 °C for 30 min with or without stirring at 1000 rpm using a plate shaker, 4% paraformaldehyde phosphate buffer solution (PFA; 100 µL) was added. After incubation at 25 °C for 20 min, fluorescent images were captured using an IN Cell Analyzer 6500 HS (General Electric Life Sciences/Cytiva, Marlborough, MA, USA). Fluorescence intensity and area of platelet aggregates were quantified using the IN Cell Developer Toolbox (General Electric Life Sciences/Cytiva).

### Platelet aggregation assay with washing in the 2D culture model

The medium was removed from the 96-well cell culture plates, and the cells were washed with PBS. Then, whole blood (80 µL/well) was dispensed into 96-well cell culture plates, and an evaluation compound (20 µL) was added. After incubation at 37 °C for 30 min with or without stirring at 1000 rpm using a plate shaker, the plate was washed thrice with PBS. Then, 4% PFA (100 µL/well) was added into 96-well cell culture plates and incubated at room temperature for 20 min. After washing thrice with PBS, 1.5% BSA in PBS (100 µL) was added and allowed to stand at room temperature for 30 min. Then, an anti-CD41 antibody with 1.5% BSA in PBS (100 µL of 10 µg/mL; MCA2245; Bio-Rad Laboratories, Hercules, CA, USA) was added and allowed to stand at room temperature for 60 min. After washing thrice with PBS, an anti-rat secondary antibody conjugated to Alexa FluorTM488 with 1.5% BSA in PBS (100 µL of 5 µg/mL; A-11006; Thermo Fisher Scientific, Waltham, MA, USA) was added and allowed to stand at room temperature for 60 min. After washing thrice with PBS, fluorescent images were captured using an IN Cell Analyzer 6500 HS (General Electric Life Sciences/Cytiva). Fluorescence intensity and area of platelet aggregates were quantified using the IN Cell Developer Toolbox.

### Platelet aggregation assay in the MPS culture model

The medium was removed from the cell culture channel of the OrganoPlate® 2-lane and washed with PBS. Then, whole blood (40 µL/channel) was added, and an evaluation compound (10 µL) was added and mixed. In the case of pre-treatment with the SYK inhibitor, whole blood was mixed with the SYK inhibitor and allowed to react for 20 min at room temperature before addition to the cell culture channel of the OrganoPlate® 2-lane. The OrganoPlate® 2-lane was placed on an interval rocker, switching between a + 7° and − 7°inclination every 3 min in a humidified incubator. Whole blood was removed, and the cells were washed three times with PBS. The plate was treated with 4% PFA (50 µL/channel) and allowed to stand at room temperature for 20 min. After washing thrice with PBS, 1.5% BSA in PBS (100 µL) was added and allowed to stand at room temperature for 30 min. Then, an anti-CD41 antibody with 1.5% BSA in PBS (100 µL of 10 µg/mL) was added and allowed to stand at room temperature for 60 min. After washing thrice with PBS, an anti-rat secondary antibody conjugated to Alexa FluorTM488 with 1.5% BSA in PBS (100 µL of 5 µg/mL) was added, and the plate was left at room temperature for 60 min. After washing thrice with PBS, fluorescent images were captured using an IN Cell Analyzer 6500 HS. Fluorescence intensity and area of platelet aggregates were quantified using the IN Cell Developer Toolbox.

### Statistical analyses

We employed the Student’s t-test to compare the means between the vehicle control group and the test article-treated group.

### Supplementary Information

Supplementary Figure 1.

## Data Availability

The data that support the findings of this study are available from the corresponding author, T.S., upon reasonable request.
