# Supplementary material for: Physiological platelet aggregation assay to mitigate drug-induced thrombocytopenia using a microphysiological system
Source: Sci Rep. 2024 Jun 19;14:14109. doi: 10.1038/s41598-024-64063-y (PMC11187140; doi:10.1038/s41598-024-64063-y)
Supplement: Supplementary file 1 — Supplementary Figure 1. [file 41598_2024_64063_MOESM1_ESM.pptx]

## Slide 1
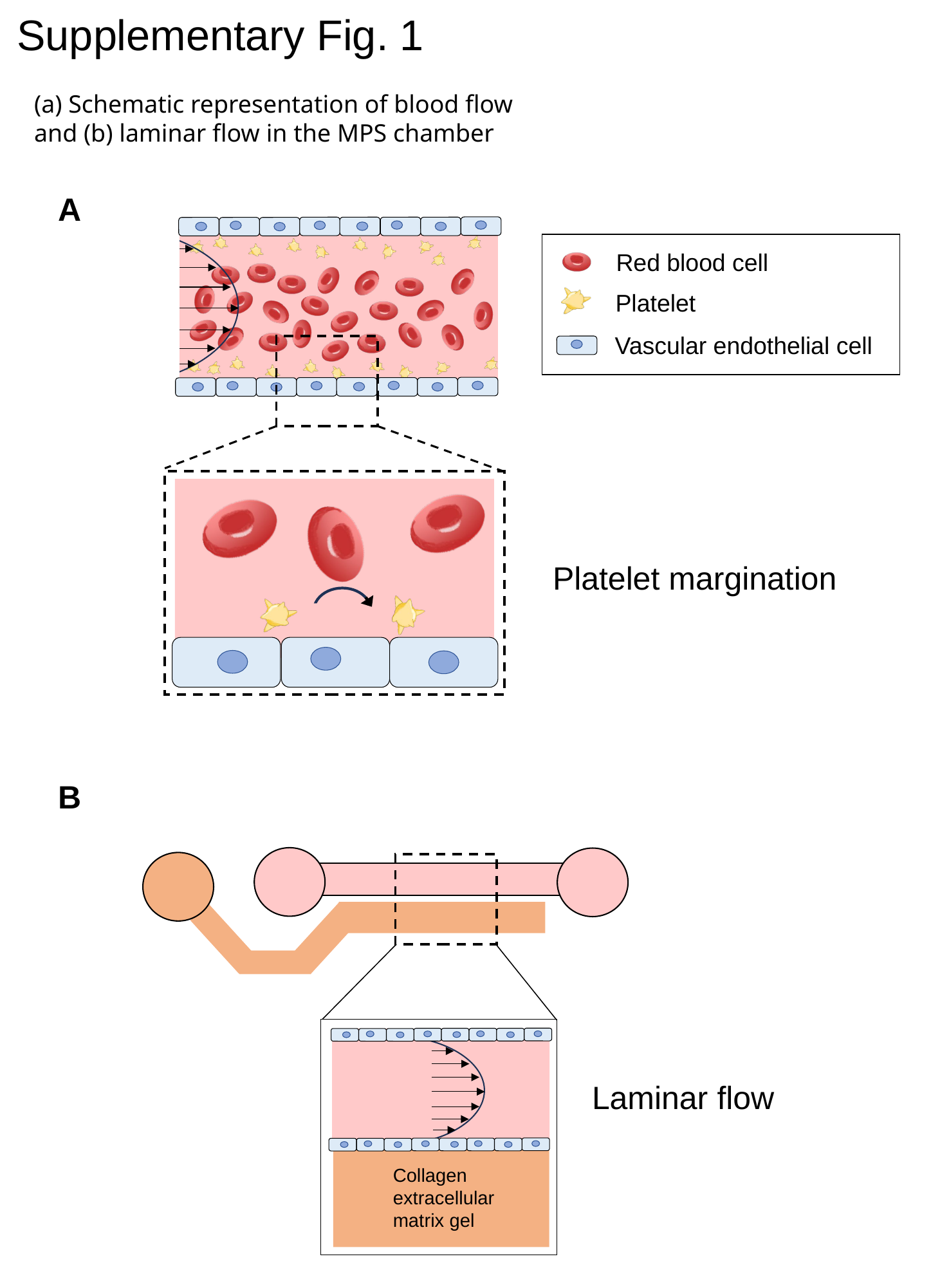

Supplementary Fig. 1
(a) Schematic representation of blood flow and (b) laminar flow in the MPS chamber
A
Red blood cell
Platelet
Vascular endothelial cell
Platelet margination
B
Laminar flow
Collagen extracellular matrix gel
